# Automated High-Throughput Quantification of Mitotic Spindle Positioning from DIC Movies of Caenorhabditis Embryos

**DOI:** 10.1371/journal.pone.0093718

**Published:** 2014-04-24

**Authors:** David Cluet, Pierre-Nicolas Stébé, Soizic Riche, Martin Spichty, Marie Delattre

**Affiliations:** Laboratory of Molecular Biology of the Cell, Ecole Normale Supérieure de Lyon, Centre National de la Recherche Scientifique, Lyon, France; Centre National de la Recherche Scientique & University of Nice Sophia-Antipolis, France

## Abstract

The mitotic spindle is a microtubule-based structure that elongates to accurately segregate chromosomes during anaphase. Its position within the cell also dictates the future cell cleavage plan, thereby determining daughter cell orientation within a tissue or cell fate adoption for polarized cells. Therefore, the mitotic spindle ensures at the same time proper cell division and developmental precision. Consequently, spindle dynamics is the matter of intensive research. Among the different cellular models that have been explored, the one-cell stage *C. elegans* embryo has been an essential and powerful system to dissect the molecular and biophysical basis of spindle elongation and positioning. Indeed, in this large and transparent cell, spindle poles (or centrosomes) can be easily detected from simple DIC microscopy by human eyes.

To perform quantitative and high-throughput analysis of spindle motion, we developed a computer program ACT for Automated-Centrosome-Tracking from DIC movies of *C. elegans* embryos. We therefore offer an alternative to the image acquisition and processing of transgenic lines expressing fluorescent spindle markers. Consequently, experiments on large sets of cells can be performed with a simple setup using inexpensive microscopes. Moreover, analysis of any mutant or wild-type backgrounds is accessible because laborious rounds of crosses with transgenic lines become unnecessary. Last, our program allows spindle detection in other nematode species, offering the same quality of DIC images but for which techniques of transgenesis are not accessible. Thus, our program also opens the way towards a quantitative evolutionary approach of spindle dynamics.

Overall, our computer program is a unique macro for the image- and movie-processing platform ImageJ. It is user-friendly and freely available under an open-source licence. ACT allows batch-wise analysis of large sets of mitosis events. Within 2 minutes, a single movie is processed and the accuracy of the automated tracking matches the precision of the human eye.

## Introduction

The *C. elegans* one-cell embryo is a large and transparent cell, very easy to manipulate and to observe under a DIC microscope. Over the past 20 years, these features combined with the power of the worm genetic, have served to decipher a large variety of essential cellular processes [1], [2]. Among those, the mechanisms controlling spindle positioning have been extensively studied both at the molecular and biophysical levels. Important discoveries were made on highly conserved molecules and mechanisms, applicable to all cells undergoing oriented cell division, including mammalian cells [3], [4].

**Figure 1:**
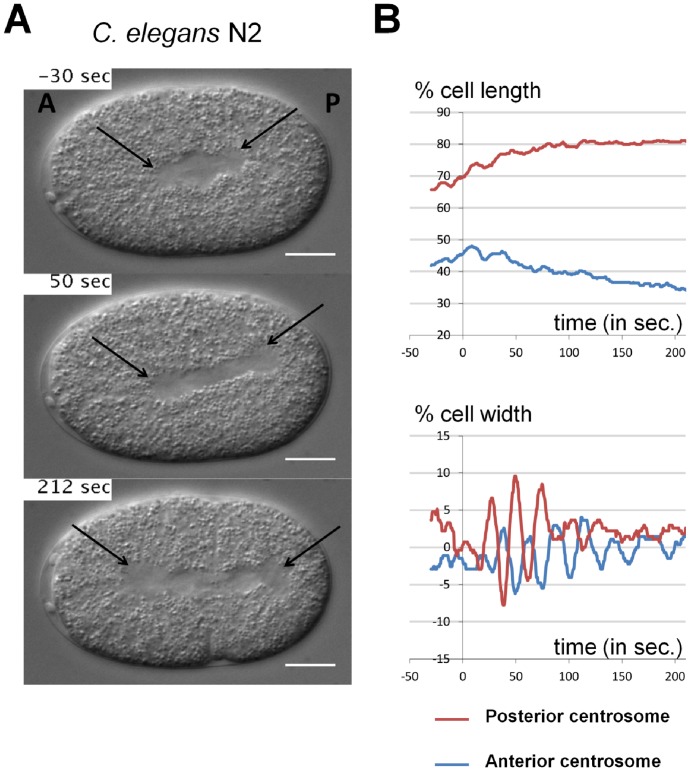
Centrosome displacements from manual tracking. **A**. Snapshots from DIC recording of *C. elegans* N2 wild-type one-cell stage embryo. Anterior is to the left and posterior to the right. Centrosomes are shown with black arrows. Scale bar is 10 µm. **B**. Manual tracking of centrosomes displacement on the transverse (upper panel) and antero-posterior (lower panel) axes over time relative to anaphase onset (t = 0 sec defined as the initial separation of chromosomes observed by DIC microscopy), for the embryos showed in A. The positions of the posterior and anterior centrosomes are represented by the red and the blue curves, respectively. For the antero-posterior displacement 0% corresponds to the anterior pole, 100% to the posterior pole of the cell. For the transverse displacement 0% is the center of the cell, 50% the upper side of the cell and −50% is the lower part of the cell.

The cytoplasm of worm embryos is filled with refringent yolk granules. As a consequence, every structure that is devoid of these granules is well visible on DIC images. Therefore, size, shape and position of the nuclei, the mitotic spindle and the spindle poles (i.e. centrosomes) can be easily detected manually (Figure 1 and Videos S1 & S2). In the one-cell *C. elegans* embryo, the mitotic spindle forms in the center of the cell and is then asymmetrically pulled towards the posterior pole of the cell. In doing so, it undergoes vigorous transverse oscillations, which reflects the mechanisms of action of force generators at the cortex [5]–[7]. The quantitative analysis of spindle displacement and oscillations in *C. elegans* embryo is therefore crucial to dissect mutant phenotypes and further unravel the physical mechanisms of spindle positioning. Analysis of spindle motion is achieved by measuring the position of each centrosome during mitosis.

Despite its short cell cycle, manual tracking of centrosomes and nuclei positions from DIC images of *C. elegans* embryos is time consuming. Therefore, the automated tracking of such objects became rapidly needed for quantitative analysis of nuclei and spindle movements. From DIC images, it has been possible to automatically track the position of nuclei, which are nicely delineated by the nuclear envelope [8]. In contrast, automated tracking of the spindle have been only developed from images of transgenic lines expressing fluorescent markers of centrosomes. These images offer an excellent signal-over-noise ratio, which facilitates automated image processing [6], [9]. Nevertheless, such analyses require establishing transgenic lines and rely on sophisticated fluorescent microscopes. Importantly, genetic analysis is then very laborious because rounds of crosses are needed to combine mutations with fluorescent reporters. Moreover, such techniques are not applicable to other worm species for which transgenesis is not accessible [10]. Last, fluorescent reporters can cause subtle phenotypic changes, introducing biases in quantitative analysis.

We therefore developed a computer program that detects centrosome position over time from DIC images. It analyzes spindle positioning quantitatively in various worm strains without requiring genetic manipulations. The computer program is a unique macro for the image- and movie-processing platform ImageJ. Within 2 minutes, movies are processed and the trajectories of the centrosomes are detected with high accuracy. The program generates a movie showing the position of centrosomes over time on the input DIC movie, which allows a visual check of the tracking quality. The program generates time-series graphs of the centrosome positions as well as a text file containing the position of each centrosome over time. Last, the program allows batch-wise analysis of large series of movies.

## Results

### Design of the ACT macro

Our macro ACT (Automated Centrosome Tracking) has been designed to help experimentalists to automatically track centrosome position during *C. elegans* mitosis, from DIC movies (Figure 1). The macro allows batch-wise movie analysis, i.e., sequentially automated tracking of multiple movies. To make the analysis most convenient for the user, the macro is divided in two parts (Figure S1): 1) A graphical user interface (GUI) loads the movies of interest and guides through the declaration of input settings that are required for the subsequent tracking process. 2) The macro launches the tracking process, movie by movie, with the specified input settings. This second part does not need any interaction with the user. Thus, the computer-time intensive tracking can be executed conveniently, at the user's discretion, in the background, over-night or on a computer cluster.

For the first part, we briefly summarize the required interactions between the user and the macro (see also Tutorial Movie S1). The GUI prompts the user to specify the path of the folder that contains all movies to be processed. Importantly, embryos must be oriented prior to the analysis, so that their anterior pole is to the left and the longest axis of the cell is horizontal. Then the GUI sequentially displays the movies and, for each of them, the user indicates the embryo size and the portion of the movie to be analyzed (starting and ending frame). Finally, the user marks the position of the centrosomes on the initial and last frame of the movie. When all movies are pre-processed, the user is prompted to launch the actual tracking.

**Figure 2:**
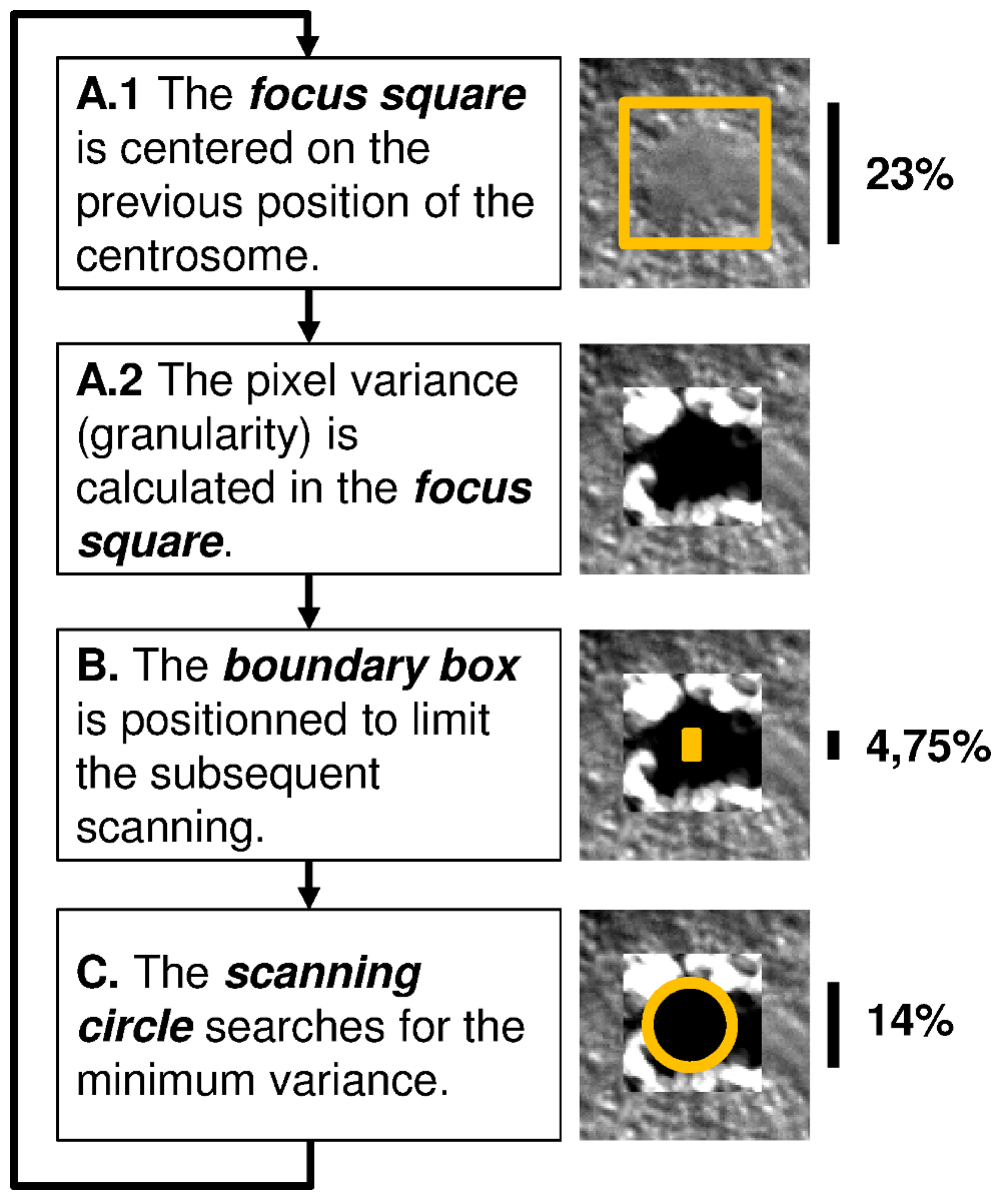
Tracking engine of the ACT macro. **A**. The position of the centrosome in the previous frame is projected on the current frame. A square window is drawn around the projected position, and the granularity (i.e., pixel-value variance within a circle of about 1% of the embryo height) is determined within this window. The macro defines the size of this focus square to be large enough for the subsequent scanning of a circular region with minimal granularity. **B**. To make this scanning process efficient (and also to avoid too strong movements of the centrosomes from one frame to another), a smaller rectangular box is defined that limits the search within the boundaries of the box. We refer to this box as the boundary box. The height (*hbb*) is about 4% of the height of the embryo. (The width of the boundary box is half of its height.) The boundary box is centered vertically on the previous centrosome position. Horizontally it is shifted either towards the anterior or posterior side of the embryo to take into account the natural dynamic behavior of centrosomes; see Table S2 for more details. **C**. The actual search is done by positioning the center of a circle (which diameter, *dsc*, is about 14% of the height of the embryo) successively to all pixels inside the boundary box. At each pixel, the mean granularity within the scanning circle is determined. The pixel for which the circle surface area shows the minimal mean granularity marks the new center of the centrosome. This position is projected to the next frame, and the tracking engine continues with step A).

In DIC movies, centrosomes are displayed as smooth circular regions with a low variance of pixel values; in contrast to their environment (the cytoplasm) that is characterized by a distinct granularity of high pixel variance. The tracking capability of the macro relies on this difference of pixel variance. To detect the position of the centrosome in a given frame of the movie, the macro's tracking engine executes five steps (Figure 2). In the first step, the position of the centrosome in the previous frame is projected on the current frame. The initial position is the one indicated by the user. Next, the tracking engine searches for a circular region of minimal mean variance in the vicinity of the projected position. To this end, rectangular boundaries are defined (Table S2) that limit the search around the projected centrosome position (the boundary box). The center of a circle is then moved within the boundary box to scan for the position of minimal mean variance (of pixel values within the circle area). When the minimum is found, the center of the scanning circle marks the new position of the centrosome in the current frame.

The ACT macro logs the tracked positions of the centrosomes for each frame into a report file (as well as additional information on the movie and input settings). It also generates a processed movie that displays an overlay of the DIC images and the detected location of both centrosomes over time (for examples, see Videos S3, S4, S5, S6, S7, S8). This movie permits rapid evaluation of the quality of the tracked trajectories. The overall tracking and reporting process requires 1–2 minutes depending on the movie length and its resolution.

### Tracking variability between human manipulators

**Figure 3:**
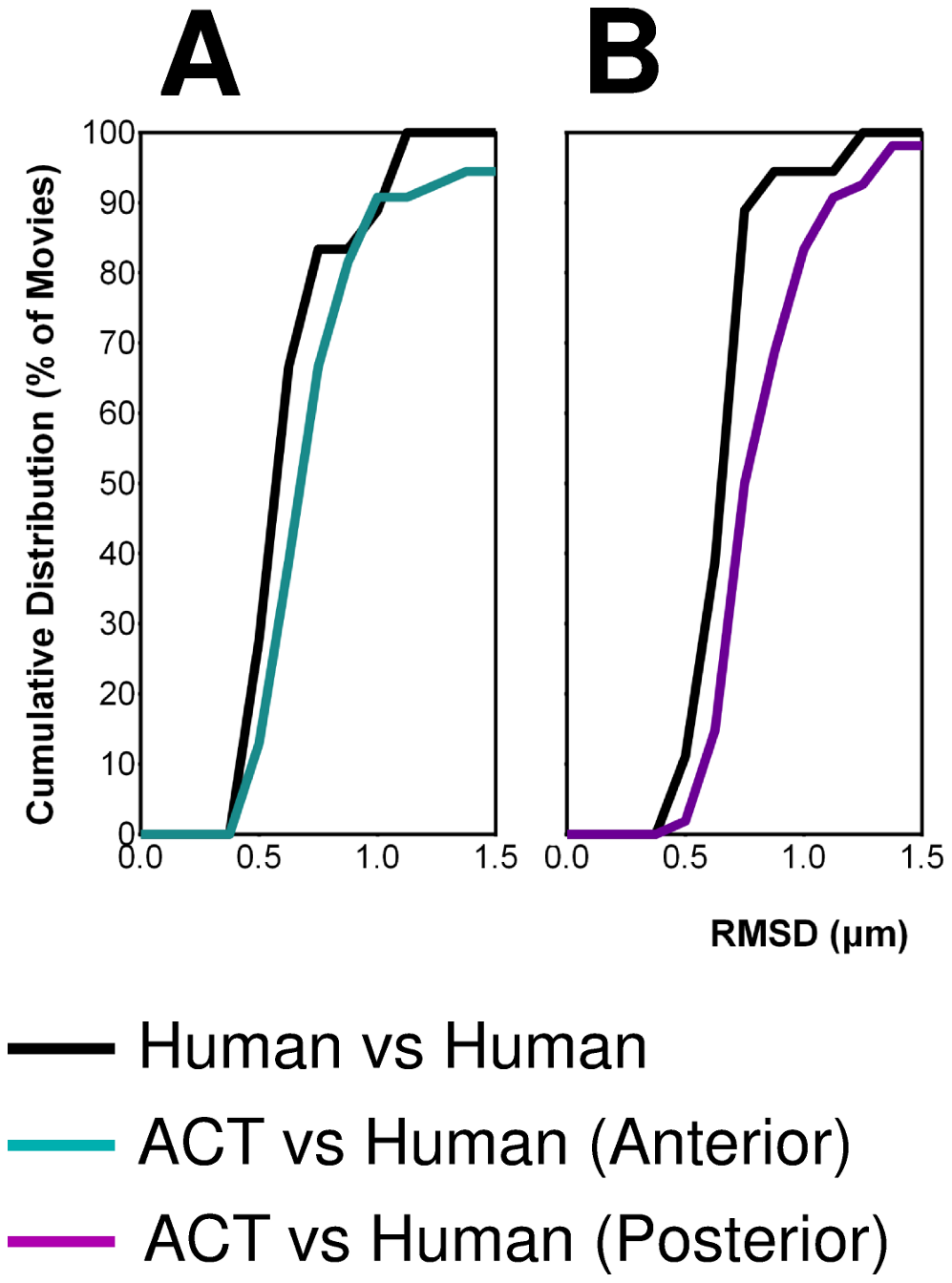
Accuracy of centrosome tracking. Normalized cumulative distributions of RMSD values for the tracked trajectories of the anterior centrosome in **A**. and posterior centrosome in **B**. The curves “Human vs Human” and “ACT vs Human” are composed of 16 and 54 movies, respectively (Table S1). The curves were constructed with a bin size of 0.1 µm.

For a critical evaluation of the macro, it was essential to know the precision of the standard procedure for manual tracking. Thus far, we performed manual centrosome tracking using the “Manual Tracking” plugin of the program ImageJ. As a control experiment, we determined the variability between different human manipulators using this plugin. Two manipulators tracked the trajectories of the anterior and posterior centrosomes for a heterogeneous collection of 18 mitosis events (Table S1). To evaluate the similarity between the detected trajectories, we measured the distribution of the root-mean-square deviation (RMSD) values for the anterior and posterior trajectories (Figure 3). RMSD is a measure for the overall deviation between two series of points (see Methods). We found that the manipulators track the centrosomes trajectories with a RMSD of 0.6±0.2 µm (i.e. 2% of the embryo height).

### Optimization of the ACT macro

In its current version, the tracking engine of the macro can be regulated by two geometric parameters: the height of the boundary box and the diameter of the scanning circle (Figure 2). In addition, we introduced a parameter to control the direction of the analysis, i.e., a logical switch that permits the tracking either in forward direction (starting from the first frame), or in backward direction (starting from the last frame).

To determine reliable settings for these parameters, we applied a classical optimization procedure, i.e., we fitted the parameter values to obtain the highest conformance with manual tracking. As a starting point for the optimization, we arbitrarily set the height of the boundary box and the diameter of the scanning circle to 1 (corresponding to 4% and 12% of the embryo height, respectively). We considered more than 160 different combinations of parameter values, by varying the relative size of the geometric parameters from 0.5 to 1.75, and by performing the analysis in forward and backward direction. We trained and tested the parameters on an extended heterogeneous set of 54 mitosis movies where the coordinates of manually tracked trajectories for the anterior and posterior centrosomes were available (including the 18 movies that were tracked by two humans). The set consisted of movies from wild-type, transgenic, mutant and RNAi treated embryos for both *C. elegans* and *C. briggsae* species, as well as wild-type strains of *C. species* 10, spanning a large variety of spindle trajectories (Table S1). We picked the optimal set of parameters through hold-out validation; the RMSD with respect to manual tracking served as scoring function (see Methods). A separate optimization was performed for the anterior and posterior centrosomes because they generally display different visual and dynamic behaviors.

Importantly, we found that the tracking of the anterior centrosome was clearly more efficient in forward direction, whereas the posterior centrosome was significantly better tracked in backward direction. We speculate that this is due to dynamic changes of the granularity within the embryo during mitosis. Among the geometric parameters, the boundary box seems more sensitive than the diameter of the scanning circle. The optimal relative height of the boundary box is 1.125 and 1.375 for the anterior and posterior centrosome, respectively. The best relative values for the diameter of the scanning circle are 1.250 and 1.125. The optimal parameter values are preset as default in the current version of the macro, but can be modified by users in the GUI.

Overall, the analysis of the 54 movies by the macro produced 6 outliers. Such outliers have an RMSD of more than 3 µm with respect to the human manipulator, and their tracked trajectories were clearly erroneous as centrosomes where detected within the nuclei (Videos S9 & S10). Causes for erroneous analyzes are given in the section “Restrictions & Recommendations”.

**Figure 4:**
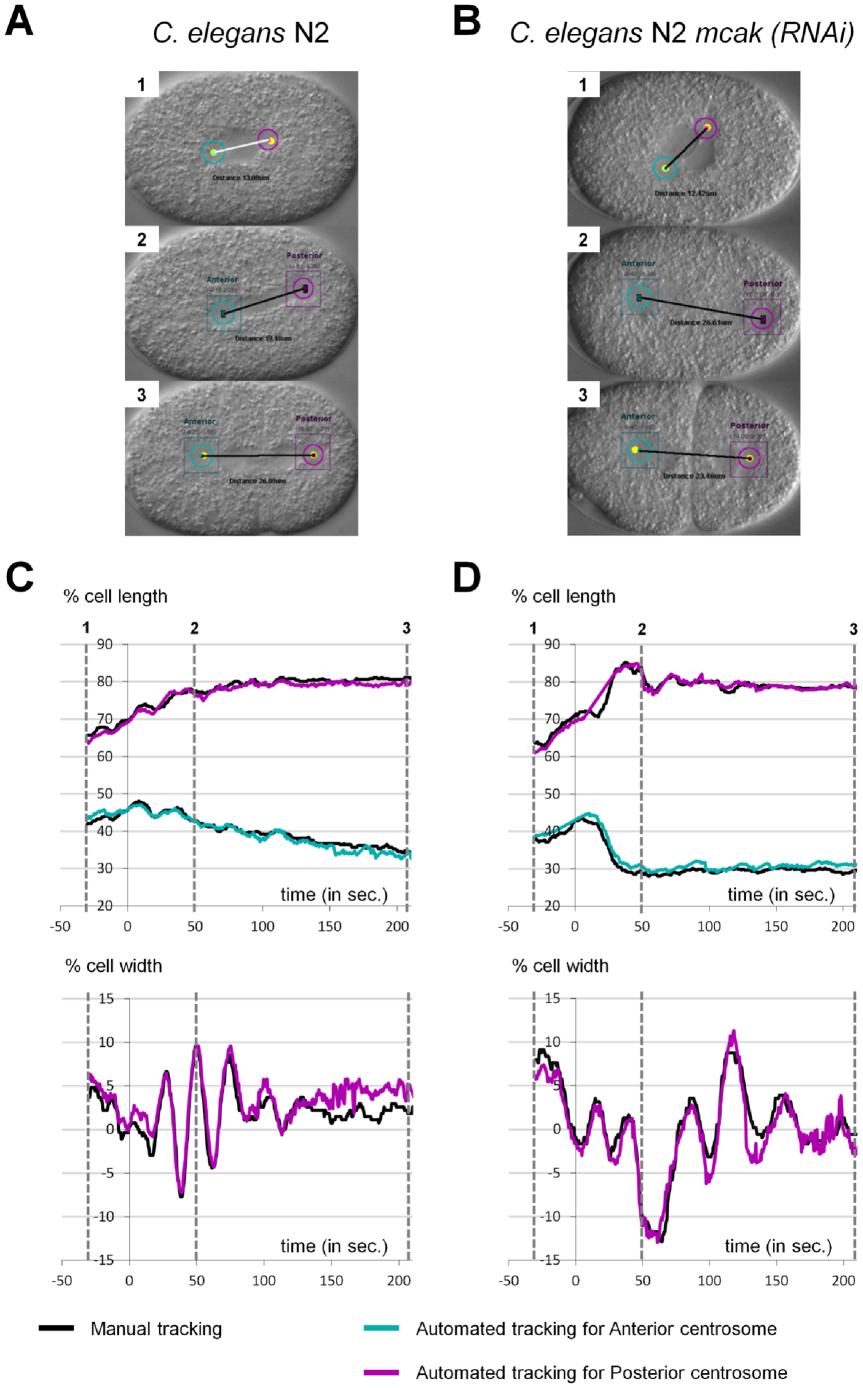
Examples of comparison between Human and ACT. **A–B**: Snapshots from output movies generated by ACT. (**A**) *C. elegans* N2 wild type, (**B**) *C. elegans* N2 *mcak-1(RNAi)* early embryos. Anterior is to the left and posterior on to right. **C–D**: Superposition of manual (black curve) and automated (cyan and magenta curves) tracking of centrosome displacements on the antero-posterior (upper panel) and transverse (lower panel) axes for the embryos presented in A and B over time relative to anaphase onset (t = 0 sec defined as the initial separation of chromosomes observed by DIC microscopy). The time corresponding to each snapshot is indicated on the graphs. For the antero-posterior displacement 0% corresponds to the anterior pole, 100% to the posterior pole of the cell. For the transverse displacement 0% is the center of the cell, 50% the upper side of the cell and −50% is the lower part of the cell.

The cumulative distribution of RMSD values (Figure 4) reveals that, apart from the outliers, the tracking accuracy of the macro matches the accuracy of the human manipulators. The mean RMSD value of “Computer vs Human” (without outliers) is shifted to higher RMSD with respect to “Human vs Human” by only 0.05 µm and 0.20 µm for the anterior and posterior centrosome, respectively. This is less than 0.6% of the embryo height, and within the error bars of the manual tracking. Overall, while a small fraction of movies (6/54) yielded totally erroneous centrosome paths, all other movies were processed with high accuracy using our macro and optimized parameters.

### Robustness of the tracking program

With the macro provided here, the user has to pick (using mouse click) the position of the centrosome centers at the beginning and at the end of the mitosis event. To investigate how the choice of the centrosome coordinates influences the tracking, we tested a large variation of different starting/ending coordinates for a given movie. We found that even with an extreme deviation of more than 1 µm at the beginning, the forward analysis of the anterior catches up quickly (within 10–20 frames) the reference tracking (i.e., the tracking with no deviation, see Figure S2). The same is true for the backward tracking of the posterior when starting with a large deviation in the last frame. Thus, the picking of the centrosome coordinates is not very crucial for the overall tracking performance. However, an extremely bad picking of the initial centrosome position may delay the convergence to the correct trajectory, reducing the exploitable portion of the tracking.

As a final test, we analyzed an extra set of 84 movies with the ACT macro (for which no manual tracking was available). The quality of the tracking was judged by visual inspection of the output movies. Out of 84 movies, 76 movies were tracked correctly and 8 gave a clear failure. The failure rate of 10% is therefore consistent with the one obtained on the 54 movies during the optimization tests.

## Discussion

We present a new and unique automated program to detect mitotic spindle positioning for high throughput projects and analyze phenotypes in a quantitative manner, from simple DIC images. With this system, data acquisition and analysis is inexpensive and fast. The time saved over manual analysis is about 10 min per movie. The program is freely available upon request. With our optimized combination of parameters, our program performs tracking accuracy comparable to the human eye for 90% of the movies analyzed.

### Restrictions & Recommendations

The macro failed to track one (or both) centrosome(s) in about 10% of the analyzed movies. We identified three reasons for this:

1. Our tracking engine is based on the identification of granularity boundaries between the centrosomes and its environment (cytoplasm, spindle). Consequently, movies cannot be tracked for the earliest steps of mitosis, i.e., when centrosomes are not expanded yet and there is no difference in granularity with respect to the spindle. At a late stage of mitosis, the posterior centrosome changes its shape and flattens. During this time period tracking of the posterior centrosome may yield to large fluctuations. Thus, the definition of the starting and ending frame is critical for the efficiency of the tracking. Our macro is therefore best suited to follow spindle trajectories from metaphase to telophase. The user should note that even if the tracking fails at the very beginning (for the posterior centrosome) or at the very end (for the anterior centrosome) of the movie, the remaining and largest fraction of the movie analysis may be still exploitable (for an example, see Videos S11 & S12).
2. The disappearance of a centrosome from the focal plan for more than 5 frames is prejudicial for the automated tracking and gives rise to an aberrant trajectory. It is therefore essential to provide constant focus during the movie. Nevertheless, it is sometimes impossible to have both centrosomes in the same focal plane, especially during the oscillation phase. Consequently, there is an increasing risk of failure to track both centrosomes in *C. elegans* mutants or other nematode species for which spindle oscillations are very pronounced.
3. Finally, the quality of the tracking is increased when movies are acquired with a strong contrast, i.e., the recorded raw DIC images should span the entire spectrum of grey levels. As a consequence, the detection of changes in granularity is more robust. Adjusting the contrast through post-processing filters is an option that will be tested in a future version.

Overall, movies with defocus and contrast problems may be still analyzed with the macro but the failure rate should increase.

Importantly, our program is robust to different pixel resolution of input movies. Indeed, it automatically scales the size of the boundary box and scanning circle to accommodate custom values of pixel resolution; the user can specify the pixel resolution in the GUI. We have tested the program's robustness for pixel resolutions ranging from 0.0645 to 0.258 µm/pixel. Lower values of pixel resolution can be used but require more RAM (>4 GB) and CPU time. Higher values than 0.129 µm/pixel may lead to inaccuracies especially for the posterior centrosome. Thus, using pixel resolution from 0.0645 to 0.129 µm/pixel does not require any reoptimization of the parameters.

Regarding the time resolution of input movies, we applied successfully the macro to movies with a frequency of 2 frames/second (as we used for the optimization process) and 10 frames/second. Lower frequencies than 2 frames/second may, however, require an increase of the boundary box parameter, *hbb*. The boundary box needs to be sufficient large to follow the movement of the centrosome. Reducing the frequency of acquisition provokes abrupt displacements of the centrosome and therefore requires a larger box. We tested a value of 0.2 frames/second for which the optimal value of *hbb* needed to be multiplied by 3. The user should be aware however that an increased value of *hbb* consumes more CPU time.

Although designed to track centrosomes in the one-cell embryo of *C. elegans*, our program can be adapted to different cell types in the worm or outside worms. We offer the possibility to change the parameters of the tracking engine, allowing users to find out the best parameters, for instance in cells where centrosomes are smaller.

Our computer program sets the stage for an accurate and objective quantification of spindle positioning from DIC movies, devoid of human variability. Its user-friendly design permits rapid high-throughput processing with tailored output generation for statistical analyses.

## Materials and Methods

### Strains and maintenance

Most strains and RNAi experiments were as described in [11]. N2 was used as the wild-type reference strain for *C. elegans* and JU1018 for *C. briggsae*. We used also two others wild-type *C. briggsae* strains: ED3036 (from China) and QR24 (from Canada). JU1333 is a wild type strains of *C. species 10* from India and JU1771 is an inbred line from JU1333 (a kind gift from MA Félix). *C. elegans gpr* mutants have been obtained from the *C. elegans* Genetic Center (CGC): VC1670 carries an homozygous deletion for *gpr-1* and RB1150 carries an homozygous deletion for *gpr-2*. Transgenic lines used are *C. briggsae* ANA037 (*unc-119(st20000)* III; *adeIs2[pMD078, Cepie-1::CeGPR-2::GFP]; mfIs42 [Cel-sid-2+Cel-myo-2::DsRed]*), *C. briggsae* ANA017 (*unc-119(st20000)* III; *adeIs3[pMD051, Cepie-1::CbGPR-2::GFP]; mfIs42* [*Cel-sid-2+Cel-myo-2::DsRed]*), *C. elegans* ANA058 (*unc-119(ed3)* III; *oxIs279[pie-1::GFP::histone]* II; *ItIs25[pAZ132, pie-1:GFP::tba-2]*), *C. elegans* XA3501 (*unc-119(ed3) ruIs32 [pie-1::GFP::H2B+unc-119(+)] III; ojIs1[pie-1::GFP::tbb-2+unc-119(+)]*) from the CGC and *C. elegans* TH251 (*unc-119(ed3) III; ddIs33[yfp::gpr-1]; unc-119(+)]*) (a kind gift from the Hyman lab). RNAi experiments were performed by feeding L3 and L4 larva for 24 to 48 h on transformed HT115 bacteria [1]. All the strains were cultured on NGM plates and fed with OP50 (*E. coli*) at 20°C, except transgenic strains that where maintained at 25°C. For one experiment JU1018 were grown at 28°C during 5 days and recorded at 28°C.

### Time-lapse DIC video microscopy

Adults were dissected in M9 medium. Eggs were collected and mounted on 2% agarose pads, covered by a coverslip and recorded at 23°C–unless otherwise stated. DIC movies were acquired using an AxioImager A2 Zeiss (objective lens 100×, NA 1.4) equipped with a Kappa camera and its accompanying time-lapse module. The resolution was 0.129 µm/pixel. Images were taken every 0,5 seconds and TIFF files were converted in Quicktime movies with the ImageJ software.

### Manual Tracking

Centrosome positions were tracked with the ImageJ software using the plugin “Manual Tracking”. Positions were determined every 0,5 seconds from the end of nuclei centration/rotation to the cytokinesis. Coordinates were saved in pixels.

### Statistical analysis

The RMSD (in µm) of a centrosome trajectory with respect to a reference trajectory (e.g., manually tracked trajectory) was calculated by
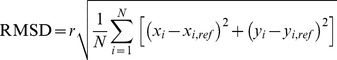
with *x_i_*, *y_i_* the coordinates (in pixels) of the centrosome at frame number *i*, *x_i_*_,ref_,*y_i_*_,ref_ the coordinates of the reference analysis, *N* the total number of frames, and *r* the resolution of the movie in µm/pixel.

The standard deviation of the RMSD for a set of movies was calculated with
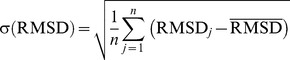
with RMSD*_j_* the value of the *j*th movie, the 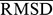 mean value, and *n* the number of movies.

### Tracking with the ACT macro

The use and installation of the ACT macro is explained in Tutorial Movies S1 and S2, respectively. As shown in Figure S1, ACT is composed of two parts. The first one is a graphical user interface guiding the scientist for movie pre-processing. It generates a command lines containing the parameters defined by the user. The second part performs the automated tracking using the previous command lines. For extensive details consult the user guide.

### Optimization of the parameters of the tracking engine

The parameters of the macro are the height of the boundary box (*hbb*) and the diameter of the scanning circle (*dsc*). In addition we implemented a parameter that controls the direction of the analysis (forward or backward). As starting point for the optimization, we made an initial guess for *hbb* and *dsc* of 4% and 12% of the embryo height, respectively. In the current version of the macro, the values of these two geometric parameters are specified relative to those initially guessed parameters (which have been set arbitrarily to 1.0).

A common strategy for parameter fitting is hold-out validation. The available data (i.e., manually tracked movies) is split into a training set (typically 2/3 of the data) and a test set (1/3). The training set is used to pick the best values of the underlying parameters, the test set serves the purpose of validation of the fitted parameters on an independent data set. We composed equally heterogeneous training and test sets of 36 and 18 movies, respectively (see Table S1). The test set corresponds to the 18 movies that have been analyzed by two human manipulators.

We performed two separate parameter optimizations for the tracking of the anterior and posterior centrosome. To evaluate the performance of the ACT macro, we defined a scoring function,
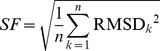
with RMSD*_k_* the root-mean-square deviation between the computer-detected and manually-tracked centrosome trajectory of the *k* ^th^ movie, and *n* the total amount of movies of the training set (*n* = 36). The goal was to minimize this scoring function by varying the values of the parameters.

We first performed a coarse-grained scan for the geometric parameters *hbb* and *dsc* where we tested all combinations of values ranging from 0.5 to 1.5 (with a step size of 0.25). This scan was done for the analysis in forward and backward direction. It turned out, that directionality parameter is extremely sensitive to the type of centrosome (anterior or posterior). Figure S3 displays the scoring function surface in the parameter space of *hbb* and *dsc* for the forward and backward analysis. For the anterior centrosome, the scoring function surface of the forward analysis is clearly shifted to lower value than the backward analysis. For the posterior centrosome the opposite is true.

Based on these preliminary results we performed a second fine-grained scan of the parameters *hbb* and *dsc* with a step size of 0.125. For *dsc*, we increased the scan to 1.75 to fully encompass the minimal region. The anterior centrosome was analyzed only in forward direction and the posterior only in backward direction. Figure S4A shows the scoring function surface of this fine-grained scan. The global minimum is found at *hbb* = 1.125, *dsc* = 1.250 for the anterior and *hbb* = 1.375, *dsc* = 1.125 for the posterior centrosome. In general, the posterior analysis does depend less strongly on the geometric parameters.

In rare cases, the macro produced outliers where the tracking engine followed a wrong particle instead of the centrosome. These outliers are usually characterized by large RMSD values (>2–3 µm) and they dominate therefore the scoring function. Since these outliers can be easily identified and removed by the user, we found it pertinent that the fitted parameters are also optimal for cases where the non-optimal analyses have been removed. We therefore investigated how the scoring function changes upon removal of the largest RMSD values. Figure S4B displays the scoring function surface for skipping the four highest RMSD values (10%). For the anterior centrosome, a new local minimum appears; the global minimum is, however, still the same. For the posterior centrosome, removal of the highest RMSD values makes the analysis even more insensitive to the geometric parameters.

To visualize the optimization gain, Figure S5A shows the distribution of RMSD values of the training set for the initial and fitted parameter values. For both, the anterior and posterior centrosome, there is a clear shift of the distribution to lower RMSD value for the fitted parameters with respect to the initially guessed parameters.

To validate the trained parameters, we applied them to the 18 movies of the test set. The RMSD distributions of the training set and test set are very similar (Figure S5B), which indicates that the parameters are not over-fitted (i.e., too sensitive towards the data).

### Supplementary Information and macro download

Two tutorial movies illustrate the installation and use of the macro. The evolution of the X,Y-coordinates is shown for the manual and computer tracking of the anterior and posterior centrosomes.

The macro, the tutorial movies, a user guide, input and processed movies can also be downloaded for our website http://www.ens-lyon.fr/LBMC/simbio/software.html

## Supporting Information

File S1 Contains 6 text files to install the ACT macro: 1) ACT_Motor CommandLine.txt, 2) ACT_Table_CommandLine_creation.txt, 3) CMD_SUM.txt, 4) Installation.txt, 5) Readme.txt, 6) LICENSE.txt.
(ZIP)

Figure S1 Architecture of the ACT macro. **A**: Graphical User Interface. The steps requiring user intervention are indicated with a specific icon and bold edge. The parameters that the user has to enter are indicated on the embryo image (3). The input and output flows are represented as dotted lines. **B**: Tracking program. Using the input parameters specified in A, the macro performs automatically the tracking for each pre-processed movie.
(TIF)

Figure S2 Robustness of the tracking engine toward the initial centrosome position. The user-detected initial position of the centrosomes was varied up to 1.2 µm (with a spacing of 0.129 µm) yielding about 348 different starting positions. For each starting position, the centrosomes were tracked with the ACT macro and the RMSD of the entire trajectory (global RMSD) was calculated with respect to the reference tracking (i.e., the tracking that started from the user-detected central position). For all 348 analyses, the tracked centrosome positions were then displayed as dots on frames 1 to 40 after the initiation of the tracking process. The dots were colored according to their global RMSD (using the color scale shown on the bottom). The percentage indicates the fraction of analyses without any deviation from the reference tracking in the corresponding frame. After 40 frames (20 seconds), even the most extreme initial deviations caught up with the reference tracking.
(TIF)

Figure S3 Scoring of the automated tracking as a function of the parameter values *hbb* and *dsc*. **A**: A coarse-grained surface of the scoring function (*SF*) is shown for the analysis in forward direction for the anterior (left) and posterior centrosome (right). The unit of *SF* is µm. **B**: Same as A but for the analysis in backward direction.
(TIF)

Figure S4 Fine-grained surface of the scoring function. **A**: The scan of parameter values for the anterior centrosome (left) was performed only in forward direction; the posterior centrosome (right) was analyzed only in backward direction. The unit of *SF* is µm. **B**: Same as A but skipping four movies with the largest deviation from the manually-tracked trajectories.
(TIF)

Figure S5Optimization of the tracking parameters of the ACT macro. The ACT macro parameters were optimized using a training set of 36 movies. The upper left panel presents the distribution of the RMSD values of the movies for the anterior centrosome with the initial (light cyan) or optimized (dark cyan) parameters. Results for the posterior centrosome are presented on the upper right panel (light magenta and dark magenta for the initial and optimized parameters respectively). The improvement of the optimized parameters on ACT accuracy was challenged on a test set composed of 18 movies. The results for anterior and posterior centrosomes are displayed in the lower left and right panel respectively. On these two graphs the RMSD distribution between human is also displayed as a black curve. The human eye sensitivity (which we defined as the mean value of the RMSD “Human vs Human” including twice the standard deviation, i.e., 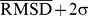) is represented as a grey dotted line on each graph. The curves were constructed with a bin size of 0.1 µm.(TIF)

Table S1 description of the 85 analyzed movies.
(TIF)

Table S2 Optimized positioning of the boundary box.
(TIF)

Tutorial Movie S1 Explains how to run ACT under ImageJ.
(MP4)

Tutorial Movie S2 Explains how to install ACT on your computer.
(MP4)

Video S1 Raw DIC movie of a wild type *C. elegans* N2 embryo. The anterior pole is to the left.
(AVI)

Video S2 Output movie of Video S1 using the ACT macro.
(AVI)

Video S3 Input movie of a *C. elegans* N2 embryo treated by RNAi against the *klp-7(mcak)* gene. The anterior pole is to the left.
(AVI)

Video S4 Output movie of Video S3 using the ACT macro, corresponding to Figure 4.
(AVI)

Video S5 Input movie of a wild-type *C. species 10* JU1333 embryo. The anterior pole is to the left.
(AVI)

Video S6 Output movie of a Video S5, using the ACT macro.
(AVI)

Video S7 Input movie of a wild-type *C. briggsae* JU1018 embryo. The anterior pole is to the left.
(AVI)

Video S8 Output movie of Video S7, using the ACT macro.
(AVI)

Video S9 Input movie used to illustrate a failed tracking. The anterior pole is to the left.
(AVI)

Video S10 Output movie of Video S9 showing a failed tracking using the ACT macro.
(AVI)

Video S11 Input movie used to illustrate a failed tracking at early steps of mitosis. The anterior pole is to the left.
(AVI)

Video S12 Output movie of Video S11 showing that the computer program failed to track centrosomes early in mitosis but eventually recovers for the rest of the movie.
(AVI)
