# Supplementary figures and images for: Automated High-Throughput Quantification of Mitotic Spindle Positioning from DIC Movies of Caenorhabditis Embryos
Source: PLoS One. 2014 Apr 24;9(4):e93718. doi: 10.1371/journal.pone.0093718 (PMC3998942; doi:10.1371/journal.pone.0093718)

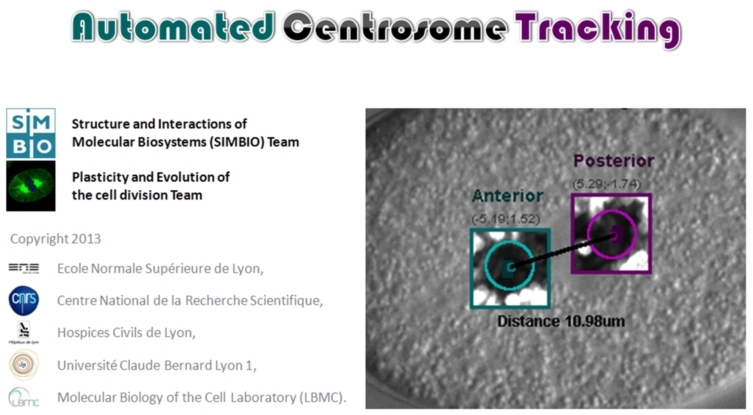

Supplement: File S1 — Contains 6 text files to install the ACT macro: 1) ACT_Motor CommandLine.txt, 2) ACT_Table_CommandLine_creation.txt, 3) CMD_SUM.txt, 4) Installation.txt, 5) Readme.txt, 6) LICENSE.txt. (ZIP) [file pone.0093718.s001.zip › ACT_CeCILL_2014-02-28/ACT.jpg]

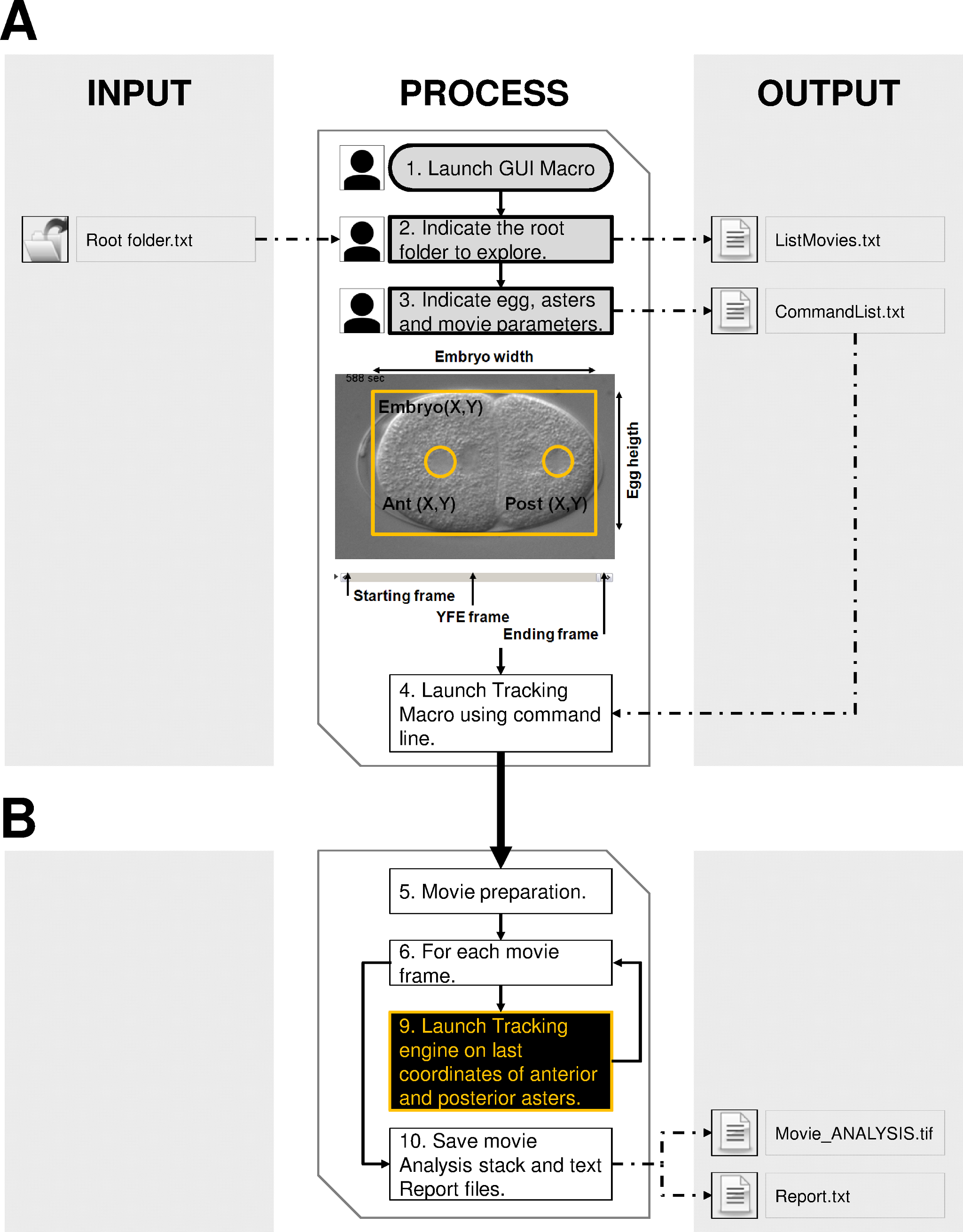

Supplement: Figure S1 — Architecture of the ACT macro. A: Graphical User Interface. The steps requiring user intervention are indicated with a specific icon and bold edge. The parameters that the user has to enter are indicated on the embryo image (3). The input and output flows are represented as dotted lines. B: Tracking program. Using the input parameters specified in A, the macro performs automatically the tracking for each pre-processed movie. (TIF) [file pone.0093718.s002.tif]

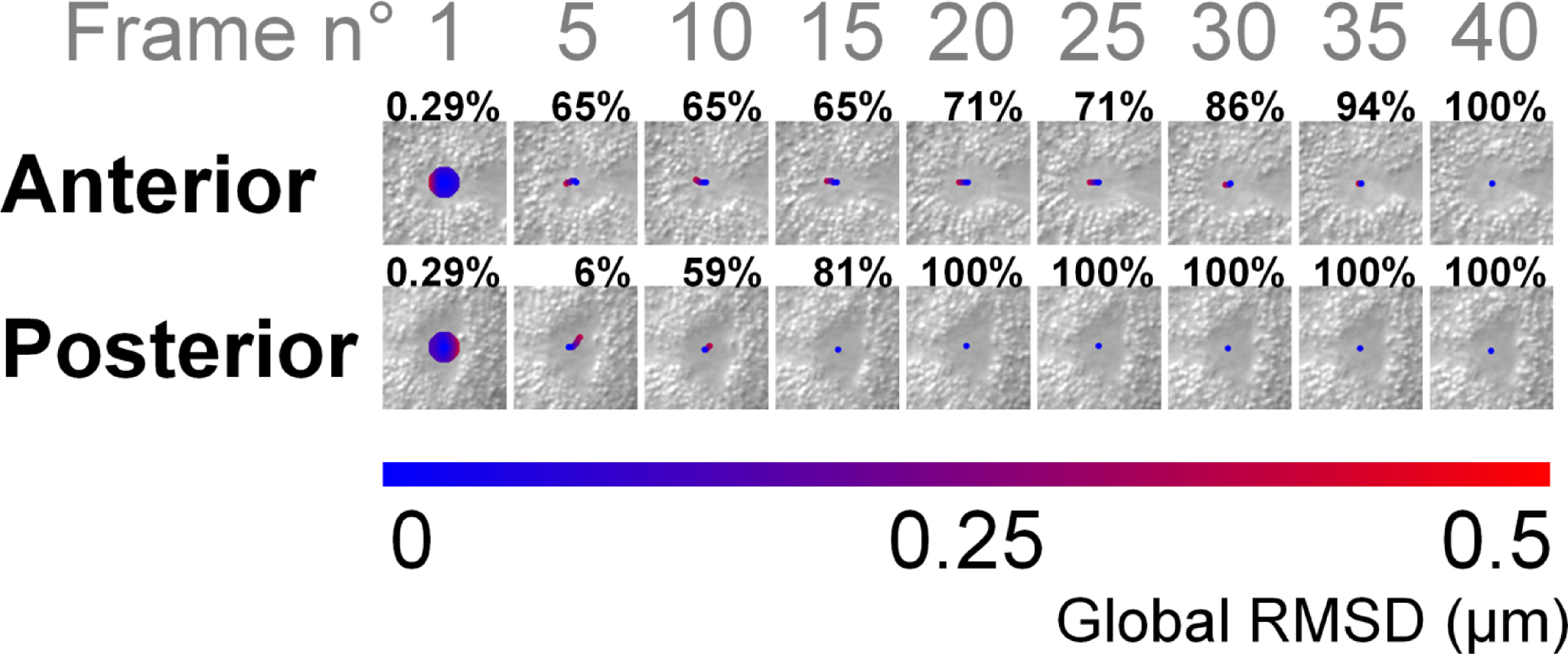

Supplement: Figure S2 — Robustness of the tracking engine toward the initial centrosome position. The user-detected initial position of the centrosomes was varied up to 1.2 µm (with a spacing of 0.129 µm) yielding about 348 different starting positions. For each starting position, the centrosomes were tracked with the ACT macro and the RMSD of the entire trajectory (global RMSD) was calculated with respect to the reference tracking (i.e., the tracking that started from the user-detected central position). For all 348 analyses, the tracked centrosome positions were then displayed as dots on frames 1 to 40 after the initiation of the tracking process. The dots were colored according to their global RMSD (using the color scale shown on the bottom). The percentage indicates the fraction of analyses without any deviation from the reference tracking in the corresponding frame. After 40 frames (20 seconds), even the most extreme initial deviations caught up with the reference tracking. (TIF) [file pone.0093718.s003.tif]

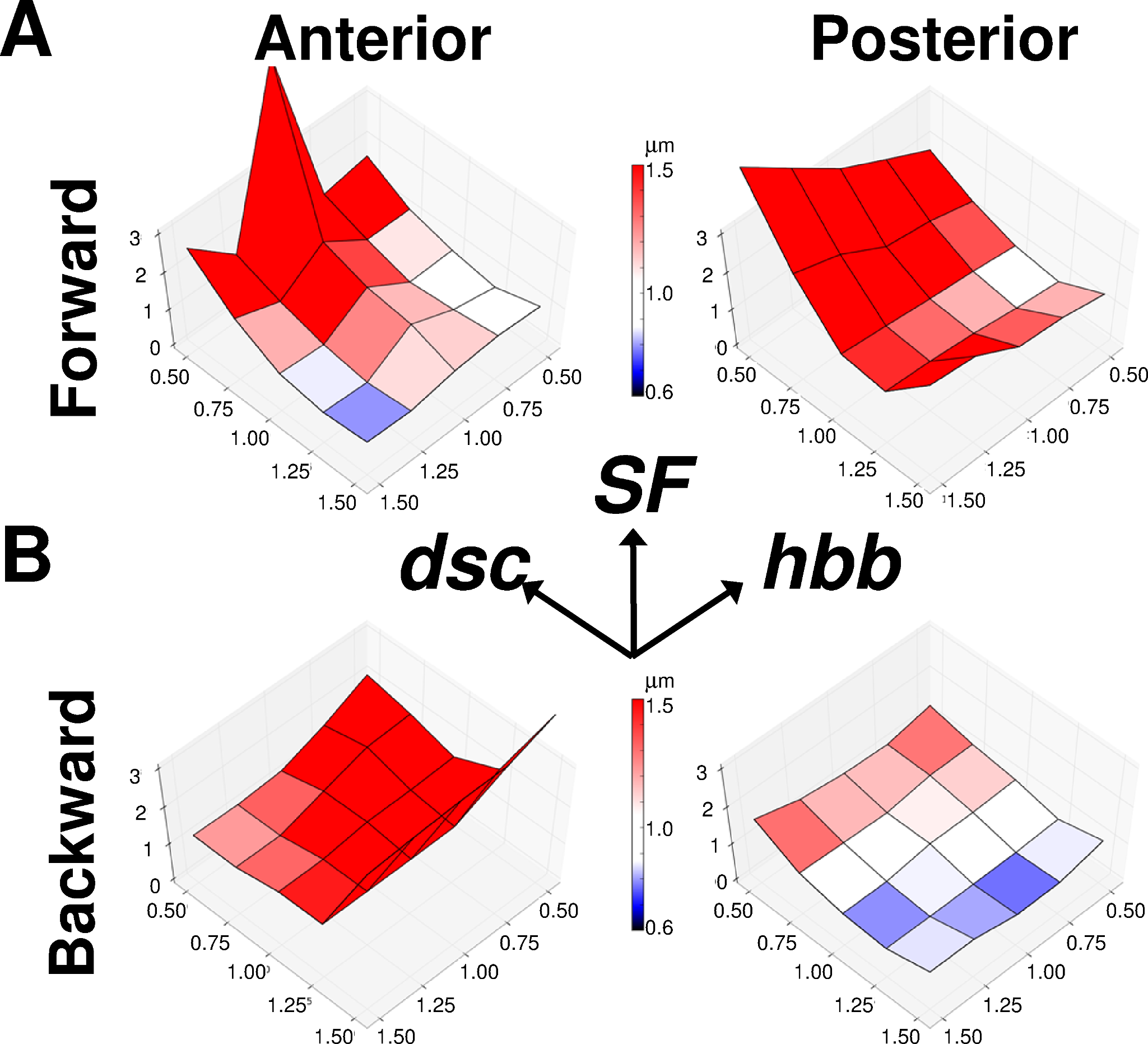

Supplement: Figure S3 — Scoring of the automated tracking as a function of the parameter values hbb and dsc. A: A coarse-grained surface of the scoring function (SF) is shown for the analysis in forward direction for the anterior (left) and posterior centrosome (right). The unit of SF is µm. B: Same as A but for the analysis in backward direction. (TIF) [file pone.0093718.s004.tif]

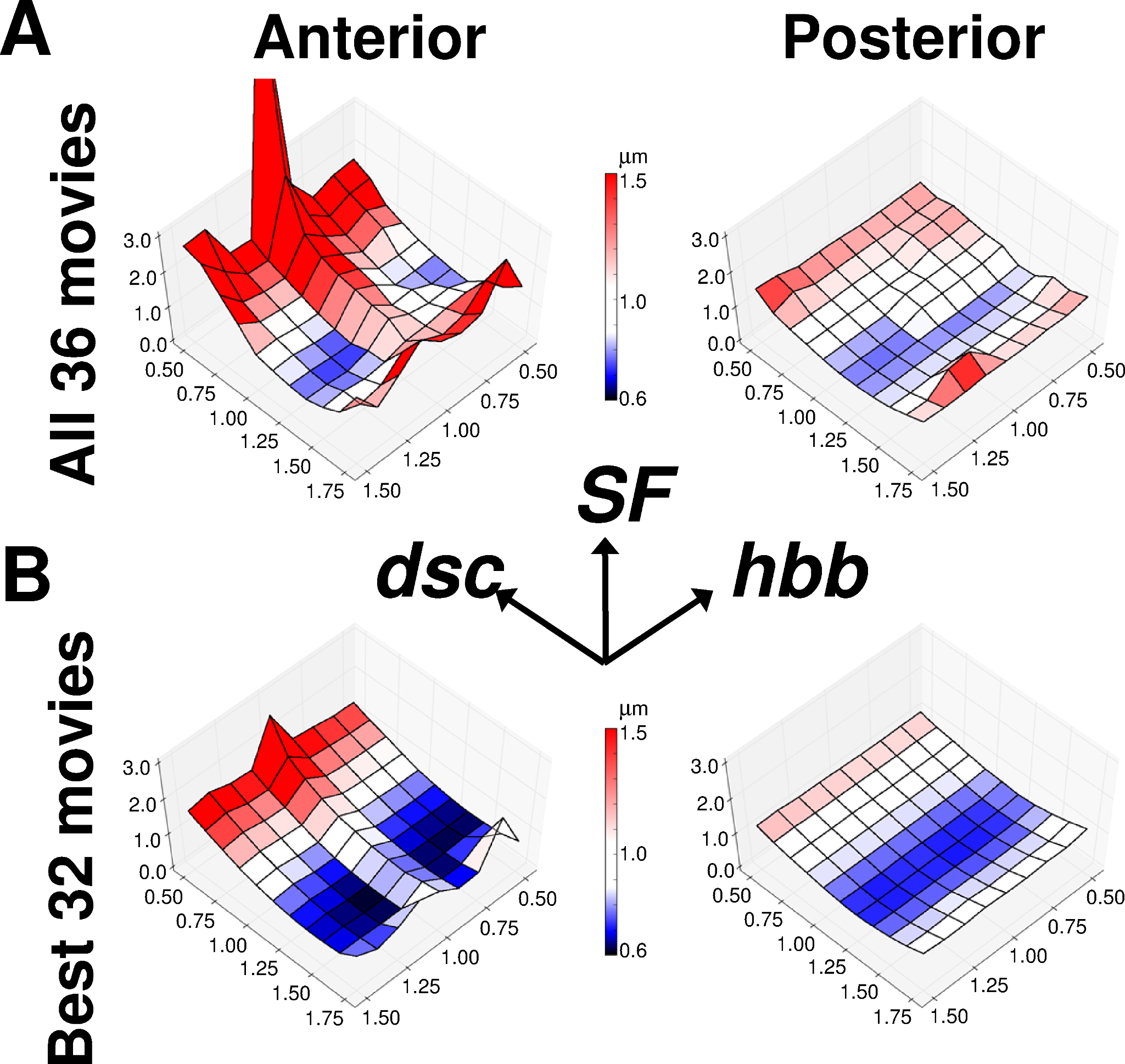

Supplement: Figure S4 — Fine-grained surface of the scoring function. A: The scan of parameter values for the anterior centrosome (left) was performed only in forward direction; the posterior centrosome (right) was analyzed only in backward direction. The unit of SF is µm. B: Same as A but skipping four movies with the largest deviation from the manually-tracked trajectories. (TIF) [file pone.0093718.s005.tif]

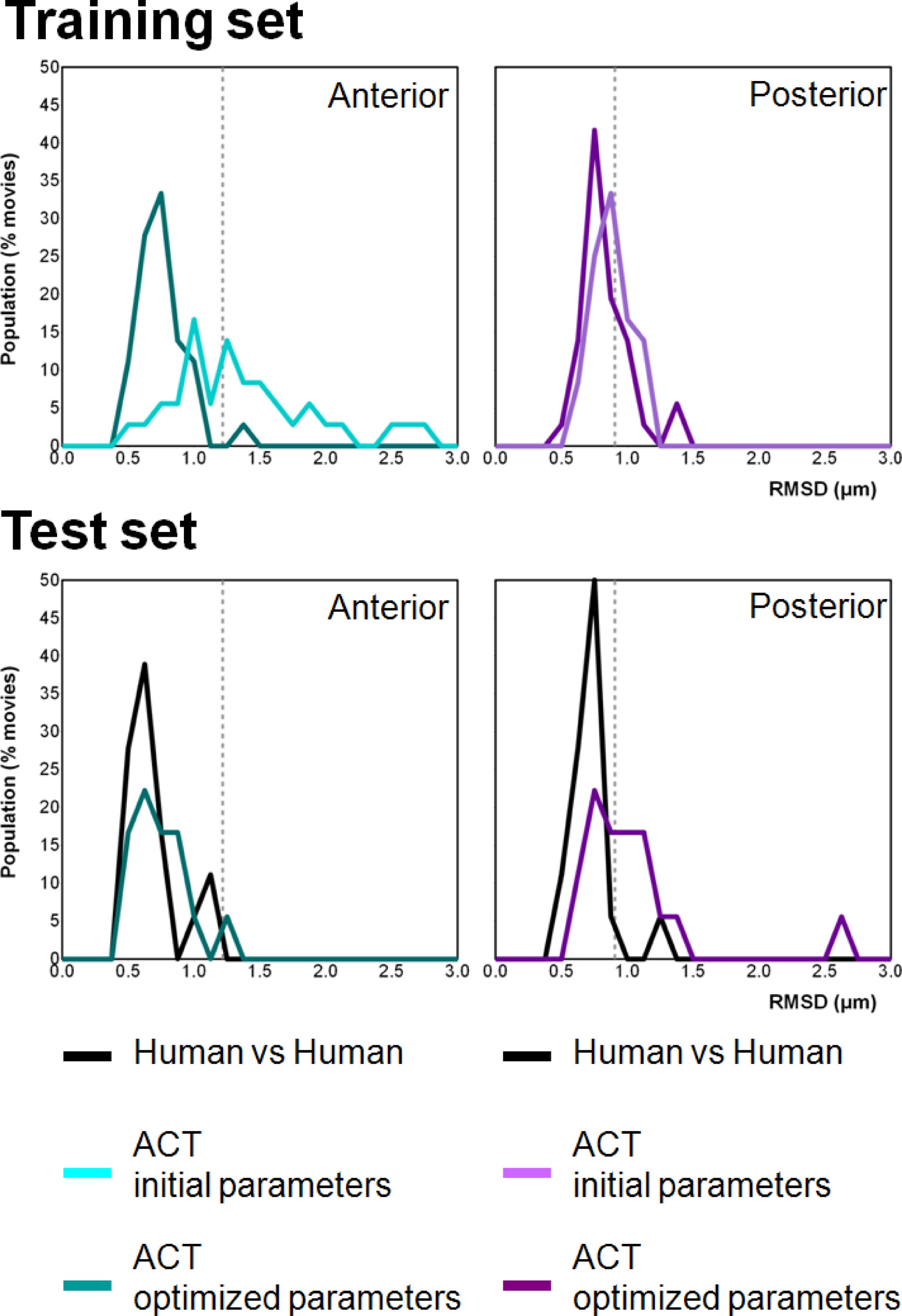

Supplement: Figure S5 — Optimization of the tracking parameters of the ACT macro. The ACT macro parameters were optimized using a training set of 36 movies. The upper left panel presents the distribution of the RMSD values of the movies for the anterior centrosome with the initial (light cyan) or optimized (dark cyan) parameters. Results for the posterior centrosome are presented on the upper right panel (light magenta and dark magenta for the initial and optimized parameters respectively). The improvement of the optimized parameters on ACT accuracy was challenged on a test set composed of 18 movies. The results for anterior and posterior centrosomes are displayed in the lower left and right panel respectively. On these two graphs the RMSD distribution between human is also displayed as a black curve. The human eye sensitivity (which we defined as the mean value of the RMSD “Human vs Human” including twice the standard deviation, i.e., ) is represented as a grey dotted line on each graph. The curves were constructed with a bin size of 0.1 µm. (TIF) [file pone.0093718.s006.tif]

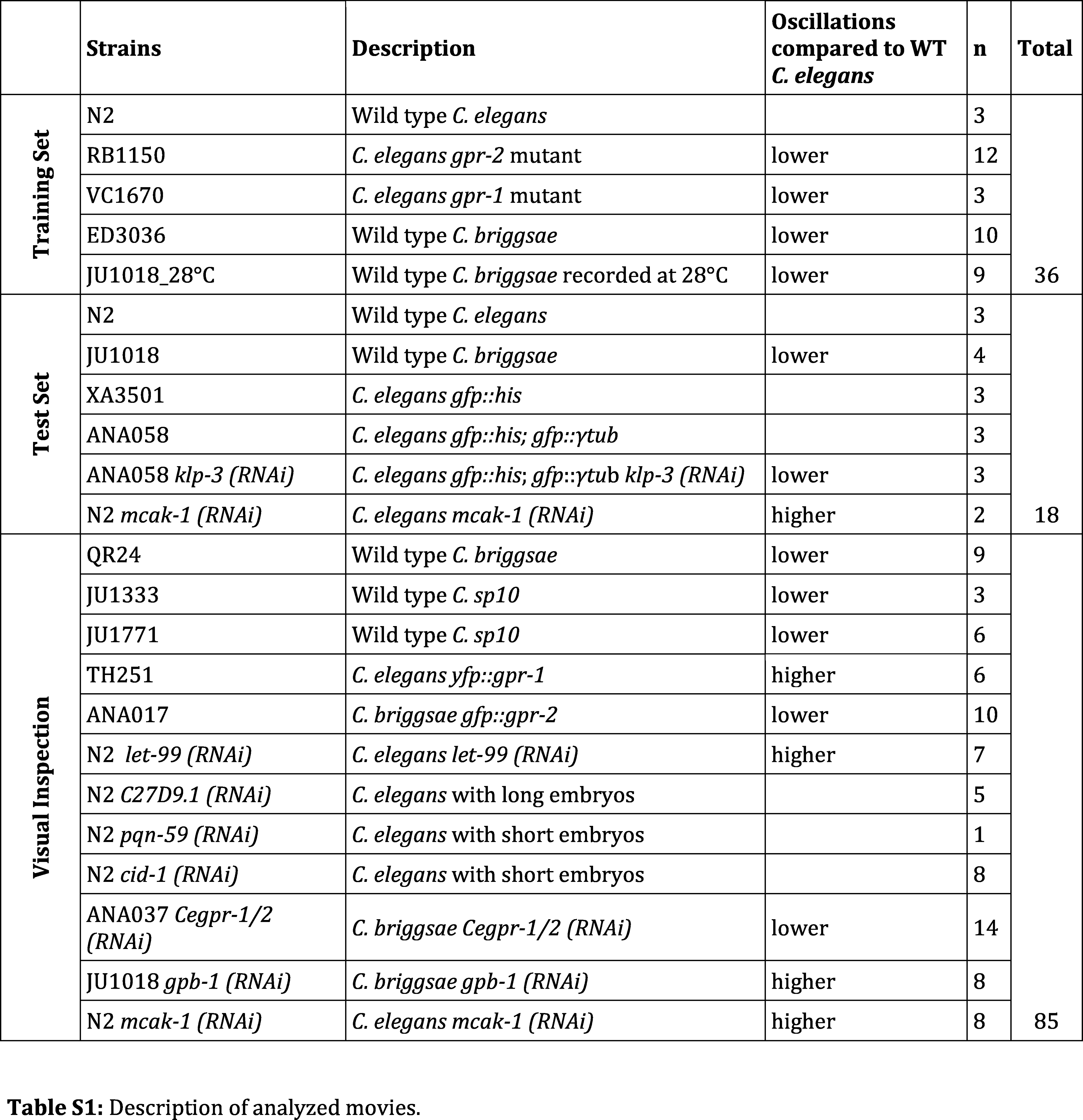

Supplement: Table S1 — description of the 85 analyzed movies. (TIF) [file pone.0093718.s007.tif]

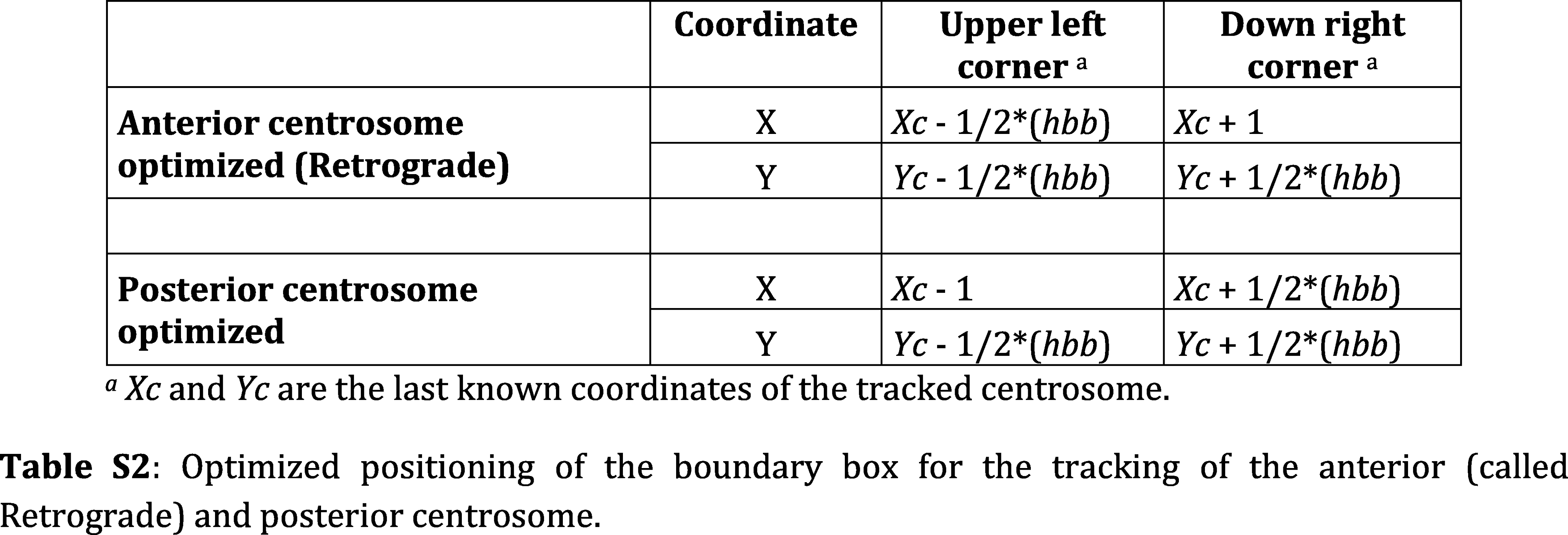

Supplement: Table S2 — Optimized positioning of the boundary box. (TIF) [file pone.0093718.s008.tif]
